# Prevalence of ExoY Activity in *Pseudomonas aeruginosa* Reference Panel Strains and Impact on Cytotoxicity in Epithelial Cells

**DOI:** 10.3389/fmicb.2021.666097

**Published:** 2021-10-04

**Authors:** Hazel Silistre, Dorothée Raoux-Barbot, Federica Mancinelli, Flora Sangouard, Alice Dupin, Alexander Belyy, Vincent Deruelle, Louis Renault, Daniel Ladant, Lhousseine Touqui, Undine Mechold

**Affiliations:** ^1^Unité de Biochimie des Interactions Macromoléculaires, Département de Biologie Structurale et Chimie, Institut Pasteur, CNRS UMR 3528, Paris, France; ^2^Université Paris-Saclay, CEA, CNRS, Institute for Integrative Biology of the Cell (I2BC), Gif-sur-Yvette, France; ^3^Mucoviscidose: Physiopathologie et Phénogénomique, Centre de Recherche Saint-Antoine (CRSA), INSERM UMR S 938, Sorbonne Université, Paris, France; ^4^Mucoviscidose et Bronchopathies Chroniques, Département Santé Globale, Institute Pasteur, Paris, France

**Keywords:** ExoY, nucleotidyl cyclase, *Pseudomonas aeruginosa*, cystic fibrosis, cGMP

## Abstract

ExoY is among the effectors that are injected by the type III secretion system (T3SS) of *Pseudomonas aeruginosa* into host cells. Inside eukaryotic cells, ExoY interacts with F-actin, which stimulates its potent nucleotidyl cyclase activity to produce cyclic nucleotide monophosphates (cNMPs). ExoY has broad substrate specificity with GTP as a preferential substrate *in vitro*. How ExoY contributes to the virulence of *P. aeruginosa* remains largely unknown. Here, we examined the prevalence of active ExoY among strains from the international *P. aeruginosa* reference panel, a collection of strains that includes environmental and clinical isolates, commonly used laboratory strains, and sequential clonal isolates from cystic fibrosis (*CF*) patients and thus represents the large diversity of this bacterial species. The ability to secrete active ExoY was determined by measuring the F-actin stimulated guanylate cyclase (GC) activity in bacterial culture supernatants. We found an overall ExoY activity prevalence of about 60% among the 40 examined strains with no significant difference between *CF* and non-*CF* isolates. In parallel, we used cellular infection models of human lung epithelial cells to compare the cytotoxic effects of isogenic reference strains expressing active ExoY or lacking the *exoY* gene. We found that *P. aeruginosa* strains lacking ExoY were in fact more cytotoxic to the epithelial cells than those secreting active ExoY. This suggests that under certain conditions, ExoY might partly alleviate the cytotoxic effects of other virulence factors of *P. aeruginosa*.

## Introduction

The nosocomial, opportunistic human pathogen *Pseudomonas aeruginosa* uses numerous secretion systems to deliver a variety of virulence factors into host cells thus contributing to establish infections. This ability together with the capacity to break down a wide range of nutrients from different environments, to out-compete co-existing bacterial species, and to develop antibiotic resistance makes *P. aeruginosa* an extremely successful pathogen (Ramos and Filloux, 2004; Pernet et al., 2014). Antibiotic resistance contributes to the mortality rate in hospital-acquired infections and pulmonary infections of cystic fibrosis (*CF*) patients (Bennett et al., 2019). During chronic lung colonization in *CF* patients, mutation rates of *P. aeruginosa* increase and strains with a mucoid phenotype that result from the overproduction of alginate emerge. In parallel, the expression of various virulence determinants, such as O antigen, pili, flagella, and phenazines decreases (Cullen and McClean, 2015). Moreover, the expression of the type III secretion system (T3SS), which enables direct delivery of virulence factors into human endothelial or epithelial cells, was also shown to change over the course of *CF* infections (Jain et al., 2004). As strains with a functional T3SS cause higher bacterial burden and mortality in acute respiratory infections (Hauser et al., 2002), the T3SS is considered as a potential therapeutic target (Lee et al., 2007; Aiello et al., 2010; Anantharajah et al., 2016; Sheremet et al., 2018).

Four different T3SS effector proteins, ExoS (49kDa), ExoT (53kDa), ExoU (72kDa), and ExoY (42kDa), can be injected through the injectisome of the T3SS into the cytosol of host cells (Yahr et al., 1997, 1998; Galle et al., 2012). Expression and secretion of the T3SS effectors are activated upon host cell contact and involve complex control mechanisms wherein the transcriptional regulator ExsA plays a central role (Yahr and Wolfgang, 2006; Williams McMackin et al., 2019).

The repertoire of accessory proteins involved in virulence including the presence or absence of a particular T3SS effector varies largely between *P. aeruginosa* strains. Most secreting strains produce no more than three out of the four effectors and usually ExoS and ExoU are mutually exclusive (Feltman et al., 2001). ExoT and ExoY appear as the two most prevalent T3SS effectors encoded in the genomes of clinical (nosocomial or chronic *CF* infections) and environmental isolates of *P. aeruginosa* (Feltman et al., 2001; Bradbury et al., 2010). Once inside the host cells, the T3SS effector proteins interact with specific eukaryotic activators to acquire enzymatic activity. The eukaryotic activator of ExoY was only recently identified as actin in its polymeric filamentous form (F-actin; Belyy et al., 2016).

The roles of the ExoS, T, and U effector proteins in infections are better understood as compared to that of ExoY. Initial studies identified ExoY as an adenylate cyclase enzyme (Yahr et al., 1998), but recent studies showed that ExoY is a nucleotidyl cyclase with broad substrate specificity (Ochoa et al., 2012). In host cells, ExoY was shown to promote the accumulation of different cyclic nucleotide monophosphates (cNMPs) with a preference for cyclic guanosine monophosphate (cGMP) and cUMP over cAMP and cCMP formation (Beckert et al., 2014), while *in vitro*, the substrate preference was shown to be: GTP>ATP≥UTP>CTP (Raoux-Barbot et al., 2018).

The contribution of ExoY to virulence of *P. aeruginosa* is still under debate. Among recent discoveries are the effect of ExoY on the host immune response involving suppression of the activation of TAK1 and decreased production of IL-1β (He et al., 2017; Jeon et al., 2017) and ExoY’s implication in converting antimicrobial amyloids into cytotoxic forms (Voth et al., 2020), a process that depends on the accumulation of extracellular active caspase-7 (Renema et al., 2020). Yet, studies investigating the effect of *exoY* deletion in *P. aeruginosa* strains in different infection models failed to reach a consensus thus far (see Morrow et al., 2017; for a recent review).

As reported by Munder et al. (2018), expression of *exoY* from a multicopy plasmid as compared to a single chromosomal copy, as found in natural *P. aeruginosa* isolates can affect the result of an experiment. One important prerequisite in studying the role of ExoY should be the knowledge of whether or not a given strain can secrete active ExoY. PCR amplification from the chromosome can only determine whether the gene is present, which is necessary but not sufficient to guarantee ExoY secretion and activity.

Here, we analyzed a set of *P. aeruginosa* strains from an international *P. aeruginosa* reference panel to assess their ability to express and secrete active ExoY by direct measurements, in bacterial culture supernatants, of the enzymatic (GC) activity of the exotoxin in the presence of F-actin as activator. In addition, we used the *P. aeruginosa* PAO1 reference strain and its isogenic deletion mutants for T3SS effectors to explore bacterial cytotoxicity in the human lung epithelial cell lines, NCI-H292 and A549.

## Materials and Methods

### Bacterial Strains and Growth Conditions

Deletion mutants of the reference strain PAO1 were constructed in the laboratory of Arne Rietsch and are listed in Table 1. *Pseudomonas aeruginosa* strains from the international *P. aeruginosa* reference panel are described elsewhere including their original references (De Soyza et al., 2013). Table 2 includes a short description indicating the source of the isolates (*CF*, non-*CF*, and early or late isolate if known) and phenotypic characteristics such as virulence in a *Galleria mellonella* infection models according to raw data obtained from Siobhán McClean, motility, alginate production, and ability to form biofilms, which were extracted from Cullen et al. (2015). Bacteria were routinely grown at 37°C with agitation in Luria-Bertani (LB) medium supplemented with 5mM EGTA and 20mM MgCl_2_ to induce the T3SS.

**Table 1:** *Pseudomonas aeruginosa* strains used in this study.

Strains	Description	References
RP1831	PAO1F	Bleves et al., 2005
RP1947	PAO1F (Δ*exoS* Δ*exoT*) (PAO1FΔ*ST*)	Sun et al., 2012
RP1924	PAO1F Δ*exoS* Δ*exoY* (PAO1FΔ*SY*)	Arne Rietsch, unpublished
RP1948	PAO1F Δ*exoT* Δ*exoY* (PAO1FΔ*TY*)	Arne Rietsch, unpublished
RP1949	PAO1F Δ*exoS* Δ*exoT* Δ*exoY* (PAO1FΔ*3TOX*)	Cisz et al., 2008
RP1923	PAO1F Δ*exoY* (PAO1FΔ*Y*)	Sun et al., 2012

**Table 2:** ExoY geno-and phenotype in *P. aeruginosa* reference panel strains.

#	Source	Strain	ExoY aa sequence^a^	*S. c.*^b^toxicity	Virulence from (1)^c^		Biofilm^d^	Motility^e^	Alginate^f^	ExoY^g^ ng/ml	GC^h^ (nmol/min/ml)	GC (nmol/min/ml/OD)
					L/H	CFU						
1	*CF*	LES B58	100		L	7.7×10^3^	++	+/−	−	150	40±1	24±0.1
2	*CF*	LES 400	100		L	2.5×10^5^	++	−	−	−	−	
3	*CF*	LES 431	100		L	6.6×10^5^	+	−	−	−	−	
4	*CF*	C3719	V158L, V320L		L	7.2×10^6^	+++	−	−	−	−	
5	*CF*, early	DK2	V158L, V320L	+	H	0.5	+++	+	−	75	33±5	23±1.5
6	*CF*	AES-1R	I69V, D110N, E181Q, V320L, and S366G		L	6.5×10^2^	++	+/−	−	−	0.6±0.05	0.7±0.04
7	*CF*	AUS23	D110N		H	2.0	+++	+	−	125	43±2	22±1
8	*CF*	AUS52	D110N, P182A		H	1.0×10^5^	+++	−	−	10	4±0.01	3.1±0.1
9	*CF*, early	AA2	P182A, S366G	+	H	0.4	+++	+	−	285	54±4	26±1.5
10	*CF*, late	AA43	P182A, S366G		H	0.6	++	+	−	530	77±1	32±1
11	*CF*, late	AA44	P182A, S366G		H	3.8	+++	+	−	560	111±3	59±2
12	*CF*, early	AMT0023-30	D110N		H	0.6	++	+	−	10	9±1	7±0.7
13	*CF*, late	AMT0023-34	D110N, frameshift P242	−	L	2.6×10^5^	+++	+/−	−			
14	*CF*, late	AMT0060-1	R193W	+	H	2.0	+++	+	−	60	27±5	18±2
15	*CF*, late	AMT0060-2	R193W		L	3.1×10^3^	+	+/−	+	−	1.1±0.2	0.7±0.2
16	*CF*, early	AMT0060-3	R193W		H	0.8	+++	+	−	90	21±0.1	12±0.2
17	Non *CF*	PAO1	100	+	H	0.3	+++	+	−	500	94±5	57±3
18	Non *CF*	UCBPP-PA14	D110N, R141Q, S227N, V320L, and frameshift F373	+	H	2.1	+++	+	−	10	0.4±0.2	0.3±0.2
19	Non *CF*	PAK	D110N, R141Q, E181Q, and V320L	+	H	1.1	+++	+	−	450	75±2	31±1
20	*CF*	CHA	D110N, E181Q, and V320L		H	2.6	+++	+	+	−	−	
22	*CF*	IST27	D110N, E181Q, and V320L		H	0.4	+++	+	+	25	15±3	11±3
23	*CF*	IST27N	D110N, E181Q, and V320L		H	0.4	+++	+	−	25	20±2	11±1.5
24	Non *CF*	968333S	100		L	2.5×10^5^	+++	−	+		9±0.1	9±0.1
25	Non *CF*	679	100		H	4.9	+++	+	−		39±3	24±1.5
26	Non *CF*	39016	L21Q, D110N, R141Q, and frameshift 242		H	0.5	+++	+	−			
27	*CF*	2192	379 codons V320L		L	1.3×10^4^	+++	+	+	−	−	
28	*CF*	NH57388A	100		L	5.1×10^3^	+++	+	−			
29	Non *CF*	1709-1	R139W, frameshift P242		H	0.4	+++	+	−			
30	Non *CF*	Mi 162	R193W, frameshift P242		H	0.8	++	+/−	−			
31	Water	Jpn 1563	V320L		H	1.9	++	+	−	25	46±1	28±3
32	Water	LMG 14084	L21Q, D110N, R141Q, and frameshift 242		H	0.3	+	+	−			
33	Hospital environment	Pr335	V320L, Q368L		H	0.8	++	+	−		29±1	18±2
34	*CF*	U018a	D110N, E181Q, and V320L		H	0.4	++	+	−		14±0.1	11±0.4
35	Tobacco plant	CPHL 9433	19 replacements	+	H	0.5	++	+	−			
36	*CF*	RP1	D110N, E181Q		H	0.3	++	+	−	150	38±17	19±9
37	Non *CF*	15108/-1	Pseudogene^i^, D110N, R141Q, G159W, R239K, and V320L		H	0.5	++	+	−	−	−	
38	Non *CF*	57P31PA	P203L		H	2.1	+++	+	−	25	28±0.2	15±1
39	Non *CF*	13121/-1	100		H	0.2	+++	+	−	450	73±1	45±6
40	Non *CF*	39177	V320L, Q168R		H	0.6	+++	+	−	110	45±1	14±0.3
41	*CF*	KK1	P182A, S366G	+	H	0.5	+++	+	−	250	60±9	29±4
42	Non *CF*	A5803	Δaa 17-20, D110N, S227N, and frameshift P242		H	1.5	++	+	−			
43	*CF*	TBCF10839	100		H	0.2	++	+	−	90	52±2	31±1.5

Empty cells correspond to values that were not determined. Cells are color coded according to Figure 2.

a 100 indicates 100% identity with the aa sequence of PAO1.

b Toxicity in *S. cerevisiae*: results according to Supplementary Figure 2; +, toxic, −, no toxicity.

c Virulence in G. mellonella according to Cullen et al. (2015) summarized as H, high virulence for LD_50_<5CFU; L, low virulence for LD_50_>650 CFU; CFU indicates average values of LD_50_.

d Values for the ability to form biofilms according to Ref (1). The authors point out that all strains formed biofilms to various degrees depending on medium and time. We reported values for the medium (MH, LB or M63) and the time point (24h, 48h, 72h) that produced maximal biofilms summarized as +, ++, or +++ for absorbance at 570 nm of <0.35, 0.35–0.95, or >0.95, respectively according to values in Supplementary Table 3 of Cullen et al. (2015).

e Motility according to Table 1 of Cullen et al. (2015); −, loss of motility; +/−, only swimming motility; +, at least 2 modes of motility detected out of 3 (swimming, swarming, twitching).

f Alginate production according to Table 1 of Cullen et al. (2015); +, production of high levels of alginate; −, low level or no alginate production.

g − Indicate protein amounts below the detection limit in western blots.

h − Indicate GC activities <0.2 nmol/min/ml.

i The exoY allele from strain #37 is annotated as pseudogene in Pseudomonas.org (2), the aa sequence aligns to that of PAO1 starting from aa 50.

### Cultivation of Human Lung Epithelial Cells

A549 human alveolar type II cell line derived from a lung adenocarcinoma (Giard et al., 1973) and NCI-H292 human bronchial cell line derived from mucoepidermoid pulmonary carcinoma (Banks-Schlegel et al., 1985) were purchased from ATCC and routinely cultivated in Dulbecco’s Modified Eagle’s Medium [(+) 4.5g/L D-glucose, (+) L-Glutamine, (−) Sodium pyruvate, and Gibco] and RPMI Medium 1640 (1x)+GlutaMAX −1 (Gibco), respectively. Culture media were supplemented with 1% L-glutamine (100x stock, 200mM, Gibco), 100units/ml of penicillin, 100μg/ml streptomycin (100x Pen Strep stock, Gibco), and 10% heat-inactivated fetal bovine serum (FBS; Sigma Aldrich) unless indicated otherwise. Cells were grown at 37°C with 5% CO_2_ supply and split when 85–100% confluency was reached. Adherent cells were detached by incubation in 0.05% Trypsin–EDTA (1x, Gibco) at 37°C with 5% CO_2_ supply for 15min maximum. Viable cells were stained with trypan blue solution (0.4% for microscopy, Sigma Aldrich) and counted using a Malassez® chamber.

### Infection of Human Lung Epithelial Cells

Around 36–48h before infection, 3×10^5^ cells/well at passage number 8–30 were seeded in 24-well tissue culture plates (Costar) in culture media with antibiotics but without the serum. Epithelial cells were infected with *P. aeruginosa* at a multiplicity of infection (MOI) 20, in culture media without antibiotics. After 5min of centrifugation at 1,000rpm to facilitate contact between bacteria and the epithelial cells for the translocation of T3SS effectors, lung epithelial cells were incubated at 37°C with 5% CO_2_ supply for 3h. Infections were stopped by the removal of the cell culture media containing bacteria and adding fresh media with antibiotics (both Pen Strep and 50μg/ml tobramycin).

### Assessment of the Bacterial Load

Infections were performed as described above with the wild-type PAO1F and Δ*Y* strains of *P. aeruginosa* in six-well (Falcon) tissue culture plates. Around 3h post-inoculation, epithelial cells were lysed to release the internalized bacteria by adding saponin (Sigma Aldrich) at 1% concentration and incubating at room temperature for 10min. Serial dilutions from the overall suspension (cell culture medium with bacteria and the epithelial cell debris+saponin) were prepared in PBS (1x, without Ca^2+^ and Mg^2+^, Sigma Aldrich), and 100μl of the 10^−4^ and 10^−5^ dilutions were spread on LB agar plates that were incubated at 37°C overnight. CFU were counted and averaged to assess the bacterial load of the wild-type PAO1F and ∆*Y* strains.

### Cytotoxicity Assays

Ten milliliter subcultures of the PAO1F, Δ*ST*, Δ*SY*, Δ*TY*, Δ*3TOX*, and Δ*Y* strains were grown to an OD_600_ of 2.0–3.0. Bacterial cultures were centrifuged at 4°C, 4,000rpm for 15min. Bacterial supernatants were discarded, pellets were washed twice with PBS (1x, without Ca^2+^ and Mg^2+^, Sigma Aldrich), and resuspended in 1ml PBS. OD_600_ 1.0 suspensions were prepared for each bacterial strain. Epithelial cells were grown and infected as described and incubated in fresh media with antibiotics at 37°C with 5% CO_2_ supply for an additional 21h.

For lactate dehydrogenase (LDH) assays, culture media containing bacteria were removed 3h post-infection, and the epithelial cells were incubated for an additional 21h in fresh medium containing the aminoglycoside antibiotic tobramycin to kill extracellular bacteria. Supernatants of infected cells were collected 24h post-infection and centrifuged at 3,000rpm at 4°C to remove detached cells. Then, the LDH from the supernatants was quantified using CytoTox 96® Non-Radioactive Cytotoxicity Assay kit (Promega) according to the manufacturer’s instructions. LDH from confluent cells equivalent to the number of infected cells per well released upon treatment with the lysis buffer provided in the kit was used as positive control based on which % of LDH release was determined.

For 7-AAD assays, supernatants of infected cells containing detached cells (i.e., potentially dead cells) were processed as follows:

Detached cells were pelleted by centrifugation at 1,600rpm for 5min and resuspended in PBA (DPBS 1%, BSA 0.1, and 0.02% NaN_3_)+7-AAD (50μg/ml) followed by incubation at 4°C in the dark for 20min. Cells were then gently washed with PBA and centrifuged again (1,600rpm, 5min). Supernatants were discarded and pellets were resuspended in 1% paraformaldehyde (PFA) and incubated at 4°C in the dark for 20min.

For attached cells, PBA+7-AAD was added to each well immediately after removal of supernatants, and cells were incubated at 4°C in the dark for 20min. Cells were then washed twice in PBA+actinomycine D (0.02mg/ml) and incubated with trypsin at 37°C for 10min. Detached cells were collected, transferred to Eppendorf tubes containing FBS for trypsin inactivation (FBS volumes were at least that of trypsin). Cells were centrifuged at 1,600rpm for 5min, supernatants were discarded, and cells were resuspended in 1% PFA and incubated at 4°C in the dark for 20min.

Finally, following the incubation in PFA, the two fractions (detached cells collected from the supernatant and attached cells) from each well were pooled and directly analyzed with a cytoFLEX, Beckman Coulter Flow Cytometer using FlowJo software.

### Light Microscopy

NCI-H292 epithelial cells were grown on plastic cover slips (Thermanox) placed in 24-well plates (Costar). After 3h infection at MOI 20, cell culture medium containing bacterial cells was discarded, adherent cells were washed twice with 1x Dulbecco’s PBS (without Ca^2+^ and Mg^2+^, Sigma Aldrich), and cover slips were mounted on 10μl Flouromount (Sigma Aldrich) drops on glass slides. Edges of the cover slips were sealed for long-term storage. Samples were imaged under the LWD 20x/0.40 objective of the Nikon Eclipse 80i microscope with a DS-U1 camera using the ACT-2U program with the following settings: exposure control: manual, AE: M, Gain: 1, DF/FL, color effect: Mono.

### Motility Assays

Swarming and swimming motilities of *P. aeruginosa* were examined as described before (Rashid and Kornberg, 2000). Briefly, 8g/L Nutrient Broth (Difco) supplemented with 0.5% D-glucose was solidified by 0.5 and 0.3% Bacto agar (Difco) for swarming and swimming motilities, respectively. Three microliter of OD_600_ 1.0 bacterial cultures were applied onto the center of agar plates and incubated at 37°C overnight. Photos were taken 24h after inoculation.

### Yeast Toxicity Assays

ExoY variants were amplified from the chromosomal DNA of the *P. aeruginosa* strains DK2, AA2, AMT0023-34, AMT0060-1, PAK, KKI, and PAO1 using oligonucleotides CATCTCGAGCGTATCGACGGGTCATCGTCAG (UM366) and GGCAGGTACCGACCTTACGTTGGAAAAAGTC (UM368). Oligonucleotides CATCTCGAGCGTATCGAACGGGTCATCGTCTG (UM367) and UM368 were used for the amplification of the *exoY* gene from the strain CPHL 9433. The resulting PCR products were digested with XhoI and KpnI enzymes and ligated into digested YEpGal555 vector (Belyy et al., 2015) for the expression under Gal1 promoter in yeast. Plasmid for the expression of the UCBPP PA14 ExoY variant was cloned previously (Belyy et al., 2018). The ExoY-encoding plasmids were transformed in *Saccharomyces cerevisiae* MH272-3fα (Peisker et al., 2008) using the lithium acetate method (Gietz and Woods, 2002). The cell viability of the resulting strains upon *exoY* expression was analyzed by a drop-test: 5-fold serial dilutions of cell suspensions were prepared from overnight agar cultures by normalizing OD_600_ measurements, then spotted onto agar plates containing yeast nitrogen base without aa (Difco), supplemented with raffinose, glucose, galactose, or their mixtures, and incubated at 30°C.

### ExoY Activity Assays From Bacterial Culture Supernatants

Fifty milliliter *P. aeruginosa* cultures were shaken at 180rpm in LB containing 5mM EGTA and 20mM MgCl_2_ at 37°C. One milliliter aliquots were taken starting from an OD_600_ of ≈0.5 as indicated, bacteria were removed by centrifugation (16,000*g*, 3min, 4°C) and supernatants were added to new tubes containing 10μl of 10% triton X-100 (to a final concentration of 0.1% triton X-100), mixed and flash frozen in liquid nitrogen before storage at −20°C for later analyses.

Samples were thawed on ice and 15μl aliquots were measured in 50μl reactions for GC activity as described previously (Belyy et al., 2016). In short: reactions containing 15μl supernatants and 3μMMg-ATP-F-actin polymerized to steady state, 50mM Tris pH 8.0, 7.5mM MgCl_2_, 0.5mg/ml BSA, 200mM NaCl, 2mM DTT, and 0.12mM ATP were preincubated for 10min at 30°C before starting the reaction by adding substrate, i.e., 5μl of GTP spiked with 0.1μCi of [α-^33^P] GTP to reach a final concentration of 2mM. Reactions were incubated for 30min at 30°C and processed as described previously (Belyy et al., 2016).

### Western Blots

Polyclonal anti-ExoY antibodies were obtained from Covalab and were raised in rabbits injected with a recombinant, truncated (aa 26–223), and C-terminally His-tagged ExoY.

Proteins were transferred to PVDF membranes using wet, manual transfers in a Mini Trans-Blot cell (BIO-RAD) for 1h at 350mA in transfer buffer containing 20% ethanol, 190mM glycine, and 25mM Tris.

Blots were blocked in PBS-Tween 0.1% (PBS-T) containing 5% skim milk for at least 1h at room temperature or overnight at 4°C, followed by two washes in PBS-T before incubation with the first antibody (anti-ExoY) diluted 1:2,000 in PBS-T containing 0.5% skim milk for 1h at room temperature. After extensive washes in PBS-T (minimum 30min and four changes), blots were incubated with the secondary antibody (ECL™ anti-rabbit-IgG horseradish peroxidase linked whole antibody from donkey, GE Healthcare) diluted 1:2,500 in PBS-T for 1h at room temperature followed by extensive washes in PBS-T. Protein bands were revealed using Pierce ECL Plus and chemiluminescent detection films (GE Healthcare). Exposure times were typically between 10s and 10min.

## Results

### ExoY Activity in Culture Supernatants of *Pseudomonas aeruginosa*

**Figure 1:**
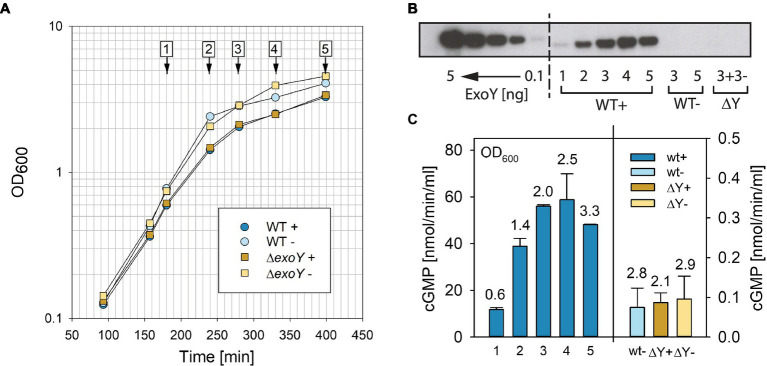
Analyses of ExoY guanylate cyclase (GC) activity in bacterial culture supernatants of the reference strain PAO1F (WT) and the isogenic Δ*exoY* mutant (Δ*Y*). **(A)** Growth curves of *Pseudomonas aeruginosa* PAO1F and the isogenic Δ*Y* mutant grown in LB in the presence of 5mM EGTA and 20mM MgCl_2_ to induce secretion of type III secretion system (T3SS) effectors (+) or under control conditions without induction (−). Numbers 1–5 represent time points where samples were taken and analyzed by **(B)** anti-ExoY Western blots and **(C)** GC activity assays. **(B)** Recombinant His-tagged ExoY or ExoY secreted from bacterial culture supernatants visualized by Western blot using polyclonal anti-ExoY antibodies against amino acid (aa) 26–223 of ExoY. Recombinant N-terminally His-tagged ExoY was used as standard and loaded at amounts of 0.1, 0.5, 1, 2.5, and 5ng. **(C)** GC activity was measured in the presence of 3μM actin polymerized to steady state in 30min reactions as described in Materials and Methods. Numbers above bars correspond to OD_600_ at which samples were taken. Values correspond to averages of at least two measurements. Error bars indicate SD.

The T3SS of *P. aeruginosa* can be induced in the presence of a metal chelator (Yahr and Frank, 1994) to express and secrete its effectors into the medium. We decided to test whether the GC activity of ExoY could be detected directly in bacterial culture supernatants in the presence of actin as cofactor to activate ExoY. We focused on the GC activity, which is the highest among the tested nucleotidyl cyclase activities with different nucleotide substrates (GTP, ATP, UTP, and CTP) using recombinant purified enzyme activated by actin (Raoux-Barbot et al., 2018). Initial experiments showed that this activity was indeed directly detectable in bacterial culture supernatants (15μl) of PAO1F without requiring sample concentration. However, prolonged sitting on ice or freezing and thawing seemed to result in substantial decline of activity. We therefore tested whether the addition of small amounts of detergent, triton X-100, could improve the reproducibility of our results. Addition of 0.1% (final concentration) triton X-100 to supernatants directly after collection from cultures by centrifugation and flash-freezing in liquid nitrogen proved to be efficient in preventing the loss of activity: no loss of activity was observed in supernatants that were repeatedly frozen and thawed for 10cycles. Figure 1A shows growth of the PAO1F wild type and the isogenic *exoY* deletion mutant (Δ*Y*) in the presence or absence of EGTA to chelate Ca^2+^ for the induction of the T3SS. Samples were analyzed by western blot for the presence of ExoY (Figure 1B) in bacterial culture supernatants. To this end, we used polyclonal antibodies against recombinant truncated ExoY (aa 26–223, C-terminal His-tag), which were raised in rabbits (Covalab). We determined the detection limit to be between 0.1 and 1ng of recombinant ExoY (C-terminal His-tag) protein. ExoY GC activities measured in bacterial supernatants are reported in Figure 1C.

### Screening *P. aeruginosa* Strains From the International *P. aeruginosa* Reference Panel for the Prevalence of ExoY

The international *P. aeruginosa* reference panel of strains (De Soyza et al., 2013) was established to represent the vast diversity of this microorganism in terms of virulence, serotype, and geographic origin. It contains commonly studied strains, clinical isolates of highly transmissible strains as well as sequential isolates from *CF* patients. The panel included initially a total of 43 strains, one of which was subsequently withdrawn from the collection (#21 corresponding to NN2). These strains were studied regarding several phenotypic traits (Cullen et al., 2015) and genome sequence (Freschi et al., 2018). For the sake of simplicity, we will refer to these strains according to the original numbering that was used in the reference article (De Soyza et al., 2013). Numbers and the corresponding strain names as well as information on virulence in a *G. mellonella* infection models according to raw data obtained from Siobhán McClean, motility, alginate production, and ability to form biofilms, which were extracted from Cullen et al. (2015) are outlined in Table 2.

As a first step to determine the prevalence of ExoY in these strains, we examined the genomic sequences of the 42 panel strains. Pairwise alignments to compare the amino acid (aa) sequences of the translated open reading frames coding for ExoY with that of ExoY from the reference strain PAO1 revealed 100% identity in eight cases (#1, 2, 3, 24, 25, 28, 39, and 43), and a number of single nucleotide polymorphisms (SNPs) leading to point mutations as reported in Table 2. In addition, we found six isolates with identical frameshift mutations that lead to truncated proteins with 248 instead of 378 aa (#13, 26, 29, 30, 32, and 42). This frameshift allele was reported previously in clinical isolates (Ajayi et al., 2003) and is illustrated in Supplementary Figure 1A (termed P242). A particular allele is represented by PA14 (#18): a frameshift in the region coding for the very C-terminus leads to a change after aa F373 and to an extended protein having 414 instead of 378 aa (Supplementary Figure 1B, termed F373). In our previous studies, we demonstrated *in vitro* using purified ExoY protein that this substitution causes a drastic reduction of the catalytic activity as the extreme C-terminus of ExoY is required for interaction with F-actin (Belyy et al., 2018). Genome analyses revealed that this mutant allele can be found in over 100 sequenced genomes (Belyy et al., 2018). Yet, in the reference panel, PA14 is the only strain harboring this allele.

ExoY was reported to have a high number of non-synonymous SNPs as compared to the other T3SS effectors (Ajayi et al., 2003). To test whether some of the SNPs affect ExoY activity, we expressed eight of these alleles (corresponding to *exoY* alleles in strains #5, 9, 13, 14, 18, 19, 35, and 41) in *S. cerevisiae* and assessed their ability to inhibit yeast growth by drop tests (Supplementary Figure 2). As previously reported (Belyy et al., 2016), expression of the *exoY* wild type allele (corresponding to the allele found in the reference strain PAO1) under the control of the GAL1 promoter in a high copy number plasmid (2μ origin) completely inhibited growth of *S. cerevisiae*. Among all tested alleles, only the allele found in strain #13 (AMT0023-34, encoding a truncated ExoY as described in Supplementary Figure 1A and termed frameshift P242) allowed normal growth (at all tested expression levels) indicating complete loss of ExoY activity. The six *P. aeruginosa* strains harboring the corresponding frameshift mutant allele (#13, 26, 29, 30, 32, and 42) were therefore assumed to be similarly devoid of ExoY nucleotidyl cyclase activity and excluded from subsequent analyses.

We then analyzed the remaining strains for the presence of ExoY GC activity in supernatants from bacterial cultures that were stimulated to secrete T3SS effectors by the addition of EGTA. To this end, cultures were grown in LB supplemented with 5mM EGTA and 20mM MgCl_2_. When bacterial cultures reached an OD_600_ of ≈ 0.5, we started sample collection in intervals of 30–60min and collected between three and six samples per strain, some of which were generally taken after cultures had reached the stationary phase of growth. Supernatants obtained after centrifugation were immediately mixed with triton-X100 (to a final concentration of 0.1%) and flash-frozen in liquid nitrogen.

**Figure 2:**
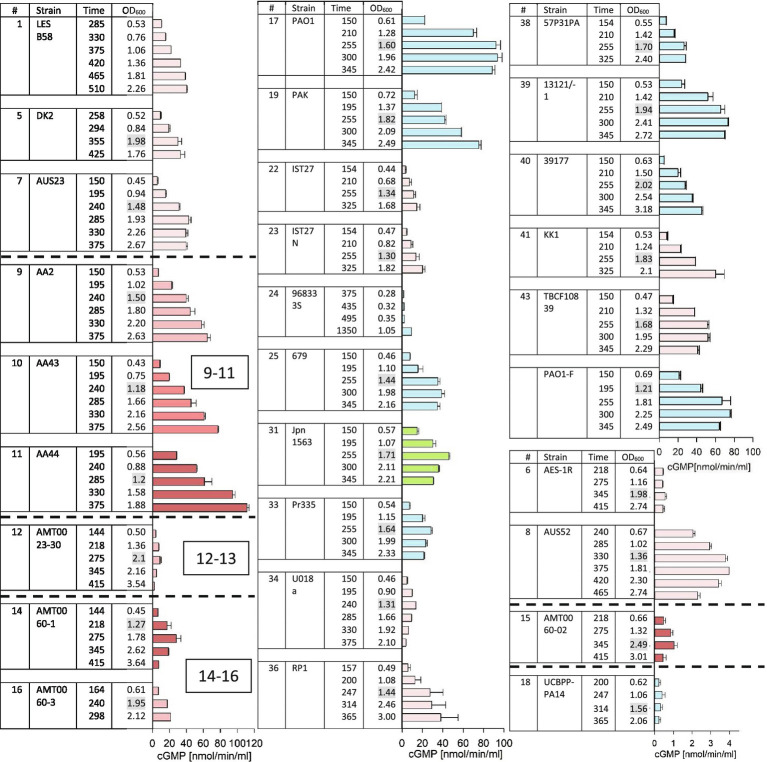
ExoY prevalence in a subset of strains from the international *P. aeruginosa* panel. GC activity was measured as described in Material and Methods in bacterial culture supernatants induced for T3SS expression with addition of EGTA. Strains that were predicted to lack ExoY activity due to frameshift P242 as described in Supplementary Figure 1 (#13, 26, 29, 30, 32, and 42) and strains that had activities below 0.2nmol/min/ml (#2, 3, 4, 20, 27, and 37) are not shown here. Cultures were inoculated (at an OD_600_ of 0.05) into LB supplemented with 5mM EGTA and 20mM MgCl_2_ to induce the T3SS. We started to collect 1ml aliquots for activity assays during the exponential growth phase starting from an OD_600_ of 0.5 and continued after bacteria had entered the stationary phase of growth. Elapsed period of time (min) since the start of cultures and OD_600_ of the collected samples are indicated in the table. OD_600_ values highlighted in grey indicate first samples that were taken after cultures had entered the stationary growth phase. GC activities were measured using 15μl of bacterial culture supernatant and correspond to averages of at least two measurements. Error bars indicate SD. Pink bars represent samples from cystic fibrosis (*CF*) isolates with darker shades corresponding to later isolates within a set of sequential isolates from the same patient. Blue bars represent non-*CF* isolates of clinical origin. Green represents the only environmental isolate among the strains with activities of ≥1nmol cGMP/min/ml.

Western blots to analyze supernatants for the presence of secreted ExoY protein were performed and are shown in Supplementary Figure 3 (for strains #3, #4, #5, #6, #7, #8, #12, #14, #15, #16, #18, #19, #20, #22, #23, #27, #36, #37, #38, and #41). Protein quantities in the supernatants were estimated by comparison with standards derived from purified recombinant ExoY protein. For those strains for which samples were analyzed by western blots, we reported one quantitative value in Table 2. This value corresponds to the peak levels that were measured among the 3–6 samples taken per strain. Fifteen microliter of culture supernatants were used for GC activity assays in 50μl reactions containing F-actin that was polymerized to steady state as cofactor necessary for the activation of ExoY. Activities varied among the samples collected for each given strain (Figure 2) and followed three different patterns: (i) continued increase of activity (ii) attaining of a plateau, and (iii) arriving at a peak after which activities dropped. Activities that reach a plateau likely reflect an arrest of enzyme expression/secretion or a continued secretion accompanied by loss of activity potentially due to enzyme degradation. A drop of GC activity could indicate that bacteria stopped producing ExoY together with changes in ExoY stability potentially due to changes in protease levels in culture supernatants as cessation of enzyme production by itself does not suffice to explain a drop in activity. We reported peak values of GC activity (nmol cGMP/min/ml of supernatant) for each given strain (corresponding to the plateau of activity or a maximum) in Table 2.

Overall, maximum values for ExoY protein amounts and GC activities in bacterial supernatants reached approximately 500–600ng/ml and 100–120nmol/min/ml, respectively with strain AA44, a late *CF* isolate as best producing strain. For the purpose of this analysis, we considered GC activities under 0.2nmol/min/ml as negative as this is close to the background activity measured in the Δ*Y* mutant strain. The international *P. aeruginosa* reference panel includes 42 strains. Two of these strains were not included in our analysis for practical reason (strains #28 and #35). Out of 40 panel strains that we analyzed, six were considered ExoY negative due to the presence of the frameshift mutation leading to protein truncation described above and in Supplementary Figure 1A, six had activities below the cut-off value of 0.2nmol/min/ml and were therefore considered negative, and three had very low activities ≤1nmol/min/ml. Among these strains was PA14 (#18 of the panel). The very low GC activity of PA14 is in agreement with our previous finding that the modified C-terminus of the ExoY variant expressed in this strain impairs interaction with actin (Belyy et al., 2018). Besides, protein quantities determined by western blots were also very low and close to the detection limit. Nevertheless, when expressed under the GAL1 promoter from a high copy number plasmid (2μ origin of replication) in yeast, this allele could cause partial growth inhibition indicating residual activity of the coded protein (Supplementary Figure 2).

Twenty five *P. aeruginosa* panel strains had activities between 2 and 120nmol/min/ml with a rather wide distribution of activities. To determine whether the prevalence of ExoY activity is different between strains originating from *CF* and non-*CF* strains, we categorized strains according to their origin. Non-*CF* strains included clinical isolates from eye and urinary infections, burn wounds, other types of pulmonary diseases as well as environmental isolates.

The mean value GC activities measured in bacterial supernatants were 26.3 and 27nmol/min/ml among the 24 *CF* and 16 non-*CF* strains, respectively. Excluding strains from the analysis that lacked GC activities; mean GC activities were 35 and 43nmol/min/ml among the 18 *CF* and 10 non-*CF* strains, respectively. Statistical analysis showed that there was no statistically relevant difference in the distribution of GC activities between *CF* and non-*CF* strains. This comparison shows that within the international *P. aeruginosa* reference panel, the presence or lack of ExoY activity is equally frequent among strains originating from *CF* patients as among the non-*CF* strains.

### Analysis of the Correlation Between ExoY Activity and Virulence of the International *P. aeruginosa* Reference Panel Strains

**Figure 3:**
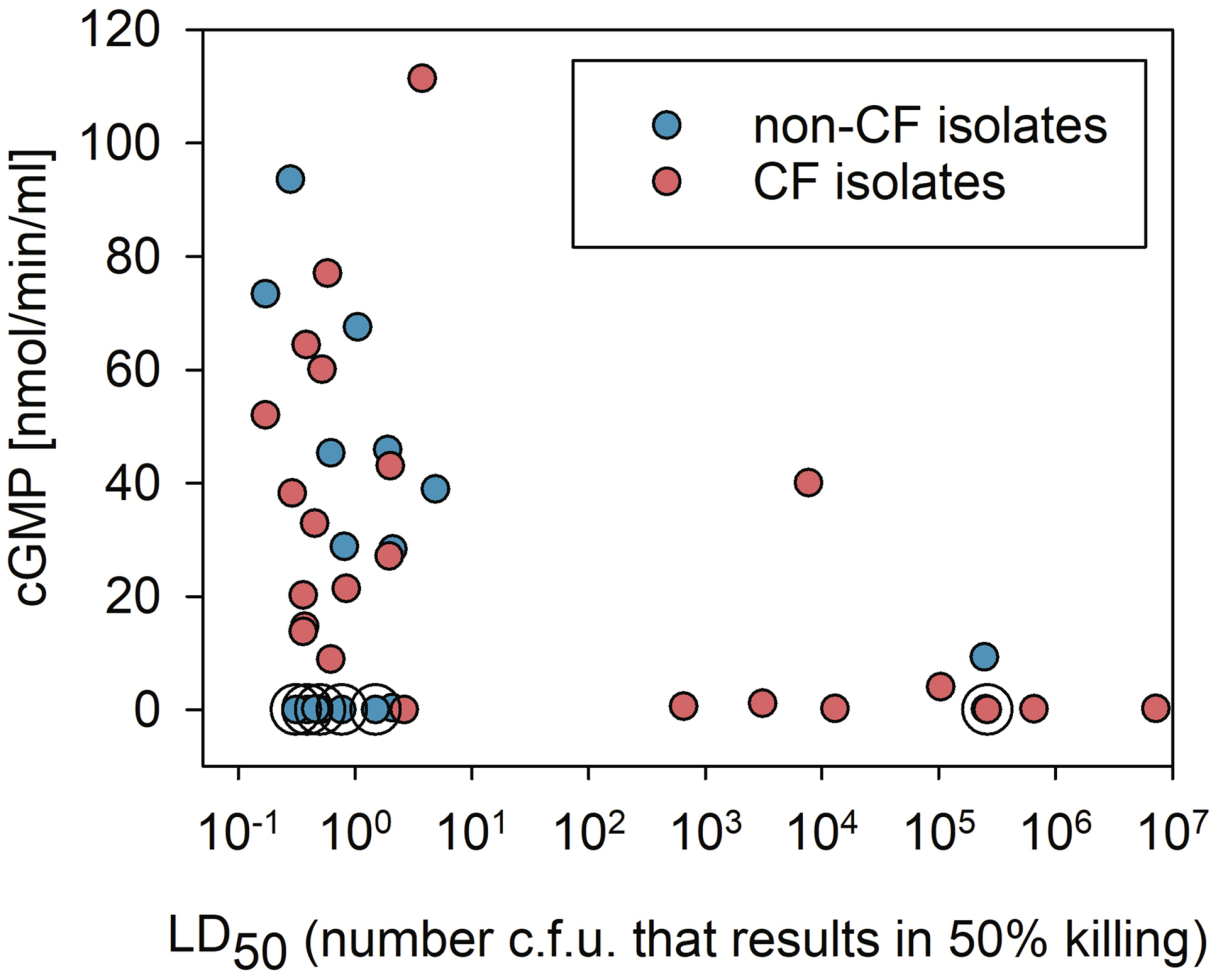
Correlation between virulence in a *Galleria mellonella* infection model (virulence data from Cullen et al., 2015) and ExoY GC activity in bacterial strains from the international *P. aeruginosa* reference panel categorized into *CF* or non-*CF* isolates. GC activities plotted correspond to the averages of peak values shown in Figure 2. Circles around data points indicate that these samples correspond to strains that were predicted to lack ExoY activity due to the presence of frameshift P242 in *exoY* as described in Supplementary Figure 1A.

Virulence of the *P. aeruginosa* reference panel strains has previously been investigated using a *G. mellonella* larvae infection model by Cullen et al. (2015). We used the data obtained by these authors to ask whether there was a correlation between the virulence of *P. aeruginosa* isolates and the presence of ExoY GC activity. In Figure 3, we plotted median lethal dose values (LD_50_, the number of bacterial CFU that killed 50% of the infected *G. mellonella* larvae) against ExoY GC activity. As shown, the virulence of the panel strains varies over a large range. In addition, we observed the following: (i) the highly virulent strains (i.e., with LD_50_ below 5CFU) showed a wide range of ExoY activities, with about 23% of these strains lacking ExoY activity (<0.2nmol/min/ml) and a mean ExoY GC activity around 44nmol/min/ml for the remaining strains (77%); (ii) most of the virulent strains not expressing ExoY were found in the group of non-*CF* isolates; (iii) all but one *CF* strains exhibiting low virulence (i.e., with LD_50_>650CFU) lacked ExoY activity; and (iv) strains capable of high ExoY GC activity, above 40nmol/min/ml, all corresponded to strains with prominent virulence (LD50<5CFU) in this infection model. Also, as previously pointed out by Cullen et al. (2015), low virulence is found more frequently in *CF* as compared to non-*CF* strains.

To analyze whether there is an overall trend/correlation between virulence and ExoY activity, we performed Spearman rank analyzes. The Spearman correlation coefficient taking all 40 strains presented in Figure 3 into account shows a weak negative correlation with a coefficient of −0.318 and *p* value of of 0.0453. This negative correlation is stronger when only *CF* strains are considered with a coefficient of −0.604 (*p* value of 0.00186). No significant correlation is found when only the 16 non-*CF* strains are taken into account (−0.00896 and 0.969 for Spearman coefficient and *p* value respectively).

### The Effect of ExoY on *P. aeruginosa* Mediated Cytotoxicity in Infected Human Lung Epithelial Cells

To study the effects of ExoY on host cells, we infected the human bronchial cell line NCI-H292 with the wild-type PAO1F strain (WT), and deletion mutants for *exoY* (Δ*Y*) or all three toxins (*exoS*, *T* and *Y*=Δ*3TOX*), and a mutant secreting ExoY as the only T3SS effector (Δ*ST*). The reference strains chosen for these experiments had a single chromosomal copy of the *exoY* gene. This experimental setup should exclude the detection of effects resulting from over-expression of ExoY from a multi-copy number plasmid (Munder et al., 2018).

**Figure 4:**
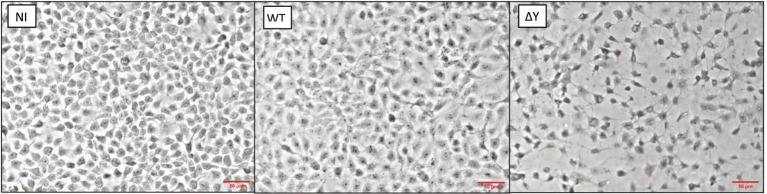
Morphology of NCI-H292 human bronchial cells infected with *P. aeruginosa* wild-type strain PAO1F or the *exoY* deletion mutant. Shown are microscopic images (obtained using a 20x objective of a Nikon Eclipse 80i microscope equipped with a DS-U1 camera) of non-infected cells (NI), cells infected with WT or the ∆*Y* mutant at an MOI of 20. Bacteria were removed 3h post-infection and processed as detailed in Materials and Methods. The experiment was performed two times and the representative images are shown. Red lines indicate the scale of 50μM.

After testing different MOI (Supplementary Figure 4), we chose an MOI of 20 for subsequent experiments. Figure 4 compares morphologies of infected NCI-H292 cells and controls. In these experiments, non-infected (NI) epithelial cells covered nearly all the growth surface of the cover slip. At 3h post-infection, whereas it was difficult to detect any morphological change with certainty in epithelial cells infected with the WT strain, the integrity of the cell monolayer of cells infected with the Δ*Y* mutant was significantly altered as epithelial cells were separated with wide intercellular gaps.

**Figure 5:**
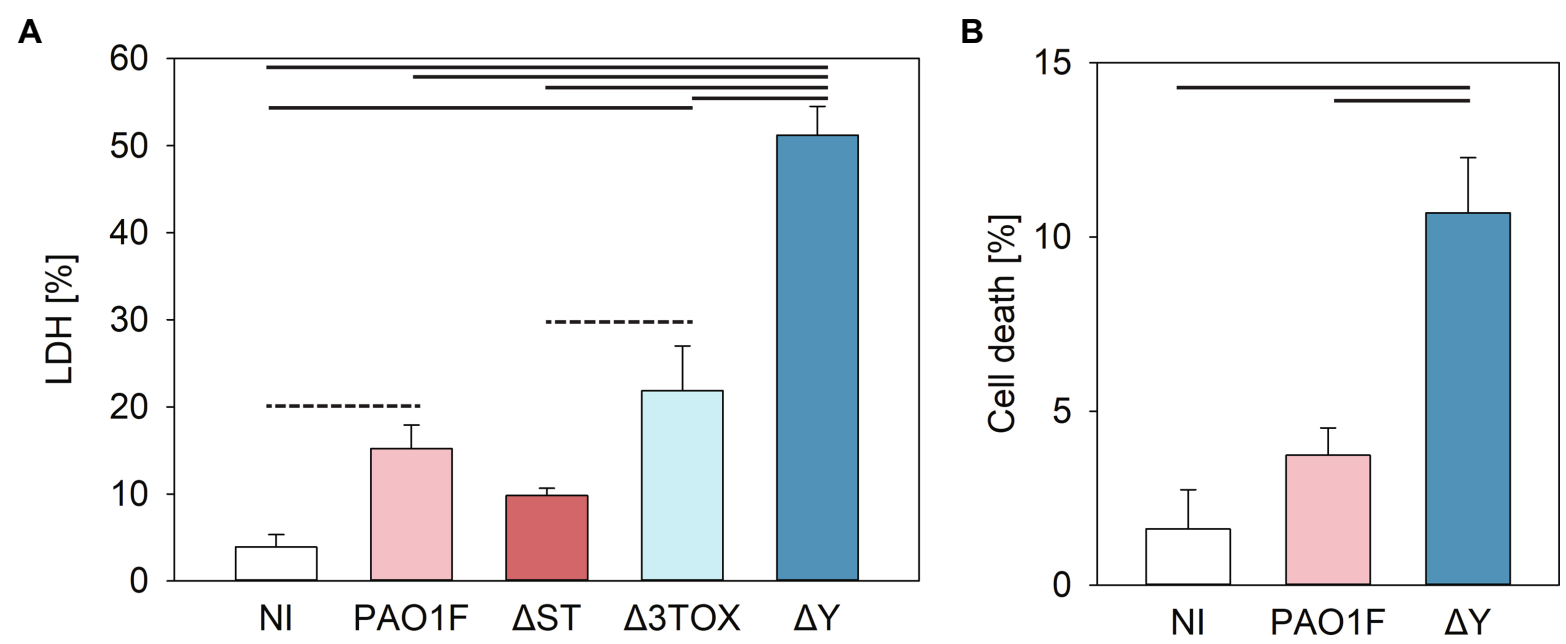
Cytotoxicity measured by lactate dehydrogenase (LDH) release **(A)** or fluorochrome 7-amino actinomycin D (7-AAD) detection of cell damage **(B)** in human bronchial NCI-H292 cells infected by *P. aeruginosa* strains for 3h at MOI 20. **(A)** Cytotoxicity was assessed based on the amount LDH released by epithelial cells into the culture supernatant measured as detailed in the Materials and Methods (determined as % of total cell LDH). Mean values and SD were calculated from three independent measurements taken using the CytoTox 96® Non-Radioactive Cytotoxicity Assay kit. One way ANOVA analysis with subsequent pairwise comparison (Turkey method) revealed significant differences between pairs connected by solid lines or dashed lines indicating value of *p*<0.001 or <0.005, respectively. **(B)** 7-AAD-mediated detection of cell damage. Cells were stained with 7-AAD as described in Materials and Methods and analyzed with a cytoFLEX Beckman Coulter Flow Cytometer and FlowJo software. Mean values and SD correspond to three independent experiments with samples measured in duplicate. Experiments were performed on NCI-H292 that had undergone between 8 and 20 passages. *Pseudomonas aeruginosa* strains expressed the following T3SS effectors: PAO1F, wild-type expressed ExoS, ExoT, and ExoY; Δ*ST* expressed only ExoY; Δ*3TOX*, expressed none of the T3SS effectors; and Δ*Y* expressed ExoS and ExoT. One way ANOVA analysis revealed significant differences (*p*<0.001) between PAO1F and Δ*Y*.

The cytotoxic effects were then quantified by measuring LDH release. NCI-H292 cells were infected with the wild-type and isogenic deletion mutants of the PAO1F strain. Our results showed that LDH release from cells infected by the Δ*Y* strain was significantly higher (~3-fold) compared to the LDH released after infection by the PAO1F, Δ*ST*, and Δ*3TOX* strains, and reached 50% of the LDH release observed in the positive control (epithelial cells treated with lysis buffer; Figure 5A).

We next examined the modulation of cell viability by ExoY using an independent method allowing the quantification of viable cells. The fluorochrome 7-amino actinomycin D (7-AAD) is excluded from live cells with intact membranes but penetrates dead or damaged cells where it binds to double stranded DNA. Staining with 7-AAD enables detection of early stages of cell toxicity. When the epithelial cells were infected with the ∆*Y* mutant, a higher number of non-viable cells were observed as compared to cells infected with the WT strain (Figure 5B). This confirms the LDH assay data and supports the hypothesis that ExoY alleviates *P. aeruginosa*-induced toxicity in epithelial cells.

We then explored whether the increased cytotoxic effects of *P. aeruginosa* lacking ExoY were also observed on human alveolar A549 epithelial cells. LDH release from the A549 cells infected by the Δ*Y* strain was again higher as compared to cells infected by the PAO1F, Δ*ST*, or Δ*3TOX* strains, although the maximal LDH release observed with A549 cells (Supplementary Figure 5) was much lower than that measured with the NCI-H292 cells indicating that the A549 cells are less sensitive to *P. aeruginosa* cytotoxicity.

**Figure 6:**
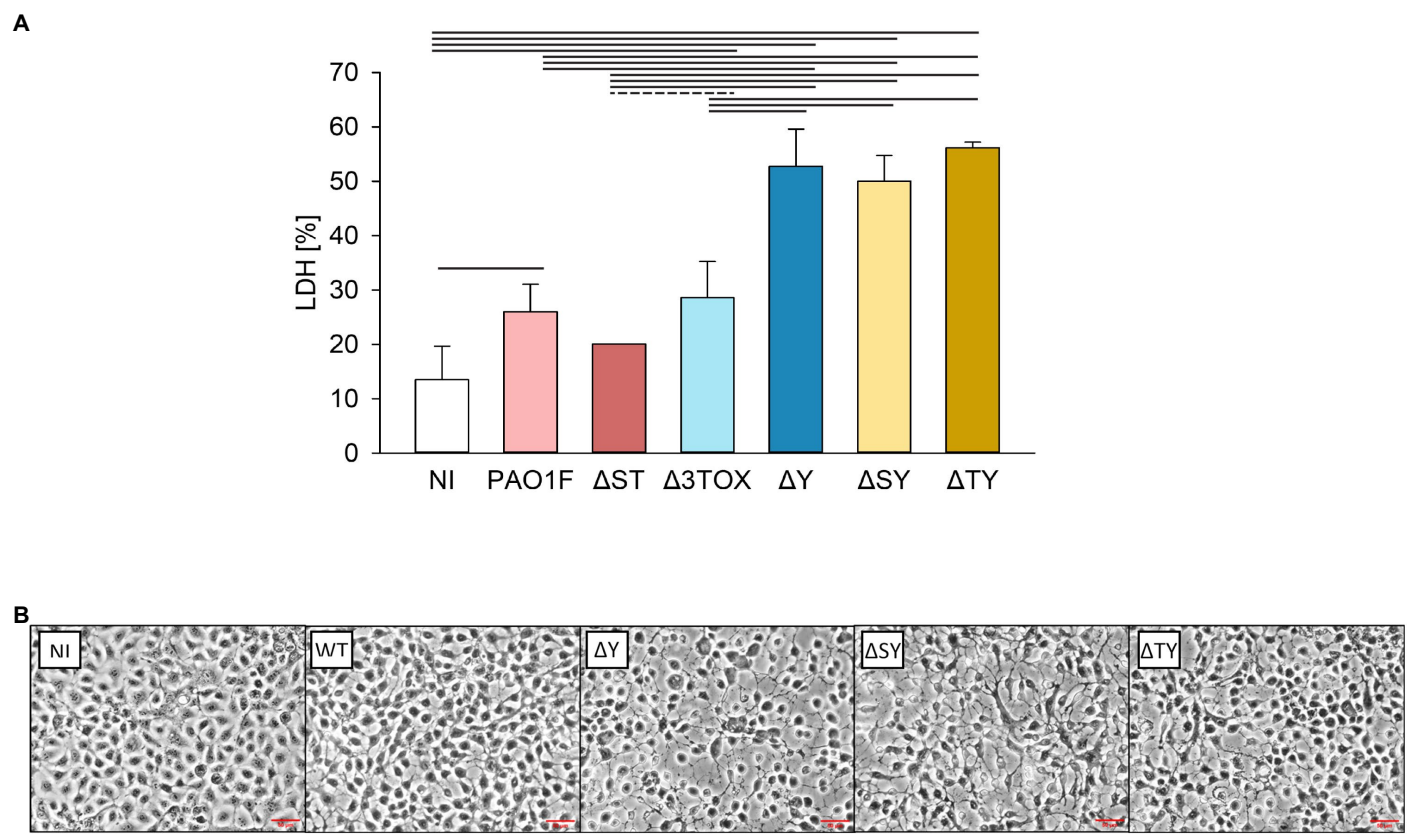
Lactate dehydrogenase release **(A)** and morphology **(B)** of lung bronchial NCI-H292 epithelial cells infected by *P. aeruginosa* T3SS exotoxin mutants for 3h at MOI 20. **(A)** Cytotoxicity was assessed based on the amount of LDH released by the epithelial cells into the culture supernatant determined as % of total cell LDH. Mean values and SD were calculated from two independent measurements with four technical replicates each taken using the CytoTox 96® Non-Radioactive Cytotoxicity Assay kit. **(B)** Microscopic images of epithelial cells infected by the *P. aeruginosa* strains. Images were taken under 20x objective using a Nikon Eclipse 80i microscope with a DS-U1 camera. The experiment was performed two times and representative images are shown. NI, non-infected control; PAO1F, wild-type (WT) expressing *exoS*, *exoT*, and *exoY*; Δ*ST*, only *exoY* expression; Δ*3TOX* no T3SS effector gene expression; Δ*Y*, *exoS* and *exoT* expression; Δ*SY*, only *exoT* expression; and Δ*TY*, only *exoS* expression. Red lines indicate the scale of 50μM. One way ANOVA analysis with subsequent pairwise comparison (Turkey method) revealed significant differences between pairs connected by solid lines or dashed lines indicating values of *p*<0.001 or 0.025, respectively.

The here described findings suggest that ExoY protects epithelial cells from the toxicity induced by other *P. aeruginosa* inherent/associated toxins. As PAO1F encodes ExoS and ExoT, we considered that those effectors could be among the sources of cytotoxicity that might be more prominent in the absence of ExoY. To test this hypothesis, we repeated the infections and cytotoxicity assays with strains expressing only *exoT* or only *exoS* (Δ*SY* and Δ*TY*, respectively). Similar levels of LDH release (~50% of the positive control) were observed after infection with the Δ*SY*, Δ*TY*, and Δ*Y* mutants indicating that they are equally cytotoxic (Figure 6A).

We next examined the changes in morphology of human bronchial NCI-H292 epithelial cells infected by these three mutants (Δ*Y*, Δ*SY*, and Δ*TY*) as compared to the WT strain using microscopy analysis. We observed signs of cell retraction and rounding with all mutants devoid of ExoY (Figure 6B). To test whether the observed differences in cell morphology and LDH release may have resulted from differences in the number of surviving bacteria in our infection experiments or differences in the motility of the Δ*Y* mutant strain, we compared bacterial loads and motilities of the WT and the Δ*Y* strains. Mean values for bacterial loads calculated from four independent experiments were 6.11×10^7^ (SD 1.87×10^7^) and 5.53×10^7^ (SD 9.13×10^6^) for the WT and Δ*Y* strain, respectively indicating equal numbers of surviving bacteria. The swimming and swarming motilities of these two strains were similar and are shown in Supplementary Figure 6.

Altogether, under the tested conditions, infections of lung epithelial cells with *P. aeruginosa* strains lacking ExoY and secreting both, ExoS and ExoT or only one of these effectors, caused greater cell retraction and cytotoxicity than that observed with the WT PAO1F strain, that secretes active ExoY. Hence, these findings indicate that ExoY somehow alleviates the *P. aeruginosa*-induced toxicity toward human lung bronchial or alveolar epithelial cells.

## Discussion

The prevalence of genes expressing T3SS effectors, among them *exoY*, was analyzed in genomes of *P. aeruginosa* isolates by PCR (Feltman et al., 2001; Bradbury et al., 2010) and *exoY* was found in the majority (98 and 89% out of 184 and 102, respectively) of clinical and environmental isolates in both studies. However, potential modifications in the gene such as the introduction of stop codons or missense mutations that might lead to loss of protein activity by truncation cannot be detected by PCR. In addition, transcription and secretion of the T3SS effectors are controlled by complex regulatory mechanisms (Yahr and Wolfgang, 2006). As a direct result, the presence of the *exoY* gene does not ensure a catalytically active and secreted protein. One of the objectives of the present study was therefore to determine the prevalence of ExoY activity among largely diverse *P. aeruginosa* isolates present in the international *P. aeruginosa* reference panel. The collection of strains ranges from clinical strains of different origin such as *CF* patients including sequential isolates from the same patient, non-*CF* clinical isolates, and strains widely studied in laboratories to environmental isolates (De Soyza et al., 2013). The consortium pursued genotypic (Freschi et al., 2018) and phenotypic characterizations (Cullen et al., 2015) of the panel strains providing valuable information that can facilitate the analysis of additional data obtained using these strains.

While type III secretion (T3S) plays an important role in acute infections (Hauser et al., 2002) where the virulence of the effectors helps the pathogen to successfully establish infections, several reports show that expression of the type-III-secreted toxins is less frequently observed in isolates from chronically infected *CF* patients: In a study by Jain et al. (2004), 435 isolates from 56 chronically infected *CF* patients were screened for ExoS, ExoT, and ExoU secretion and only 12% of the strains were found to secrete at least two of these exotoxins. Comparison with environmental isolates and newly infected patients indicated that strains change from a T3S-positive to a T3S-negative phenotype in *CF* patients. An additional study including more than 2,500 *P. aeruginosa* isolates from 114 *CF* patients confirmed this tendency (Jain et al., 2008). The lower percentage of T3S-positive isolates indicates that the toxicity of the effectors might be incompatible with a lifestyle adapted to chronic infections.

ExoY was not investigated in the above-mentioned studies. However, one study, performed on *P. aeruginosa* strains isolated from patients with hospital-acquired pneumonia (using western blots to detect the presence of the protein), showed that among the 35 tested strains, 78% secreted ExoY (Schulert et al., 2003). In addition, a recent study genotyping *P. aeruginosa* isolates from 99 critically ill, mechanically ventilated patients, showed a 93% prevalence of the *exoY* gene. GC activity was verified *in vitro* from six of the *exoY* encoding *P. aeruginosa* isolates by measuring cGMP accumulation in infected cells (Wagener et al., 2020).

In our study, not unexpectedly, the overall prevalence of ExoY activity was lower as compared to that of the presence of the *exoY* gene: whereas all 40 strains harbored the gene, only 25 out of 40 strains (62%) secreted active ExoY (considering GC activities above 1nmol/min/ml). Furthermore, we found no significant difference in ExoY secretion between *CF* and non-*CF* strains.

Among the sets of sequential isolates from chronic lung infections of *CF* patients, ExoY expression varied between the isolates of each of the three sets (#9–11, #12–13, and #14–16). The observed variations indicate that ExoY activity levels do not inevitably decrease with the duration of *P. aeruginosa* lung colonization and can even increase as seen for strains #9–11.

We used metal chelation by the addition of EGTA to the growth medium to induce expression and secretion of ExoY into the bacterial culture medium. It is not known how this artificial setup reflects the *in vivo* conditions where contact between bacteria and human cells are needed for induction. Some of the strains might not be inducible *in vitro* but might be responsive to cell contact or inducibility might depend on the growth milieu. On the other hand, a feedback regulation involving ExoS was documented (Cisz et al., 2008) and might self-limit T3SS expression including that of ExoY. Thus, our *in vitro* setup could also lead to overestimation of ExoY activities. Therefore, we consider that strains that secreted active ExoY into the culture medium in our study have the capability to produce ExoY *in vivo*, while this activity can be modulated by multiple factors.

The role of ExoY in pathogenesis remains poorly understood and the effects of ExoY on target cells are still a matter of controversy (see Morrow et al., 2017; for a recent review). Expression of ExoY is highly toxic in yeast cells or transfected HeLa cells (Belyy et al., 2016), and ExoY was shown in several studies to have severe effects in infection models (Morrow et al., 2017). Additionally, we observed that, among strains from the international *P. aeruginosa* reference panel, those that exhibited high ExoY GC activity (i.e., above 40nmol/min/ml) also showed prominent virulence (LD50<5CFU) in *G. mellonella* infection model (Figure 3). Yet, despite these observations, we found here that the presence of ExoY could also potentially counteract the cytotoxicity of *P. aeruginosa* caused by other virulence factors. Under the conditions we used, the deletion of *exoY* resulted in increased cytotoxicity toward human lung epithelial cells. A protective effect of ExoY was not seen in previous studies, which used different cell types, strains, or infection conditions than those presented here (Lee et al., 2005; Vance et al., 2005; He et al., 2017). However, in a study performed by Huber et al. (2014), ExoY was shown to induce a slight spreading when endothelial cells (HUVEC) were infected as opposed to ExoS/T which causes cell retraction through their effect on the actin cytoskeleton. This study implicated inactivation of Rho, Rac, and Cd42 GTPases and alteration of the Lim kinase-cofillin pathway in the effect on the actin cytoskeleton, which eventually results in cell retraction/rounding. An ExoY-mediated stabilizing effect on the actin cytoskeleton is in agreement with our *in vitro* data showing that (i) ExoY binding along actin filaments stabilizes them and inhibits their severing and disassembly catalyzed by Actin-depolymerizing factor (ADF)/cofilin proteins and (ii) the cell content of F-actin increases in ExoY-transfected epithelial cells as compared to that of non-transfected cells (Belyy et al., 2016). These data are consistent with a potential protective effect of ExoY against cytotoxicity caused by ExoS/T. The interplay between co-injected effectors could be affected by the concentration of the individual effectors in the host cell, localization, activities, and those could in turn be manipulated by the host cell response leading to either amplification or dampening of their effects in the host. Synergistic and antagonistic interactions between co-delivered effectors were described in different bacterial pathogens (Ichikawa et al., 2005; Cain et al., 2008; Woida and Satchell, 2020). The intricate interplay between co-injected effectors can of course also be affected by growth conditions in the laboratory, strain background, and infection model systems. An example illustrating this, was described by Lee et al. (2005) who used an artificially constructed PAK strain expressing all four effectors (ExoS, T, Y, and U), and showed that depending on the inoculum, co-expression of ExoS, T, and Y can either diminish or augment the virulence caused by ExoU in a mouse virulence model. We believe that these seemingly contradictory observations may in fact indicate that additional complex regulatory mechanisms operate *in vivo*.

Based on our data showing that ExoY counteracted the overall cytotoxicity of *P. aeruginosa* toward human epithelial cells, we thus conclude that, ExoY can possibly exert a protective role at certain stages of bacterial infection to facilitate host colonization or to establish and/or maintain chronic infection of the host. In addition, the present work showed that the GC activity of ExoY can be measured directly in bacterial supernatants. This allowed us to analyze *P. aeruginosa* strains from the international reference panel for their ability to secrete active ExoY protein. There remains much to learn about the role of ExoY in infections and its coordination with the other T3SS effectors and *P. aeruginosa* virulence factors. The ability to detect ExoY presence and activity in different strains will facilitate this challenging endeavour.

## Data Availability Statement

The original contributions presented in the study are included in the article/Supplementary Material; further inquiries can be directed to the corresponding author.

## Author’s Note

As reported here, the CHA strain (#20) from the *P. aeruginosa* panel lacked ExoY activity in our assays. However, we recently obtained from an independent source another CHA strain that we found to secrete active ExoY. This discrepancy might be related to potential variation over time as the strain circulated in the scientific community.

## Author Contributions

UM, DL, LR, LT, and HS contributed to conception and design of the study. HS designed and performed experiments and data analysis for results of Figures 4, 5A, 6, and Supplementary Figure 4–6, as well as preliminary experiments for Figure 2, and wrote the first draft as well as sections of the second draft. FM, FS, DR designed and performed experiments and data analysis for Figures 1, 2, Supplementary Figure 3, and Table 2. AD and AB designed and performed experiments and data analysis for Figure 5B and Supplementary Figure 2, respectively. VD designed and performed experiments and data analysis for Figure 6. UM designed experiments for Figures 1, 2, designed and performed experiments for Table 2, data analysis for Figures 1, 2, 3, Supplementary Figure 1, and Table 2, and wrote the second draft. UM, DL, LR, LT, HS and AB were involved in editing the second draft. All authors contributed to manuscript revision, read, and approved the submitted version.

## Funding

This project was funded by the Institute Pasteur under #PTR 43-16, by the ANR under ANR-18-CE44-0004, and by CNRS UMR 3528 and the Air Liquide Foundation and Association des Motards du Viaduc de Millau (France). AB was supported by a stipend from the Pasteur – Paris University (PPU) International PhD Program.

## Conflict of Interest

The authors declare that the research was conducted in the absence of any commercial or financial relationships that could be construed as a potential conflict of interest.

## Publisher’s Note

All claims expressed in this article are solely those of the authors and do not necessarily represent those of their affiliated organizations, or those of the publisher, the editors and the reviewers. Any product that may be evaluated in this article, or claim that may be made by its manufacturer, is not guaranteed or endorsed by the publisher.

## Nomenclature

AA: Amino acid
7-AAD: Fluorochrome 7-amino actinomycin D
CF: Cystic fibrosis
GC: Guanylate cyclase
LDH: Lactate dehydrogenase
T3SS: Type III secretion system
T3S: Type III secretion
WT: Wild type.
