# Supplementary material for: Prevalence of ExoY Activity in Pseudomonas aeruginosa Reference Panel Strains and Impact on Cytotoxicity in Epithelial Cells
Source: Front Microbiol. 2021 Oct 4;12:666097. doi: 10.3389/fmicb.2021.666097 (PMC8524455; doi:10.3389/fmicb.2021.666097)
Supplement: Supplementary file 1 [file Data_Sheet_1.pdf]

Supplementary Material

Supplementary Figures

A

Frameshift P242

```
Mi162 aa241  ·T··P·  ·S··V··Q··R··I··P··*·  aa248
             accccc-tcggtgcaaaggatcccctgagcgaggacggattctatggcagggaggatatg
             |||||| |||||||||||||||||||||||||||||||||||||||||||||||||||||
             acccccctcggtgcaaaggatcccctgagcgaggacggattctatggcagggaggatatg PAO1
PAO1  aa241  ·T··P··L··G··A··K··D··P··L··S··E··D··G··F··Y··G··R··E··D··M· aa260
                                                                          of 378
```

B

Frameshift F373

```
PA14 aa361  ·V··P··L··R··P··S··F··Q··E··A··L··D·  ·F··S··N··V··R··S··D··A· aa380
PA14  1081 gtgccgctgcggcccagcttccaggaggcgcycgac-ttttccaacgtaaggtctgatgcg
           |||||||||||||||||||||||||||||||||||| ||||||||||||||||||||
PAO1  1081 gtgccgctgcggcccagcttccaggaggcgctcgacttttttccaacgtaaggtctga
PAO1 aa361  ·V··P··L··R··P··S··F··Q··E··A··L··D··F··F··Q··R··K··V··*·   aa378


PAA14 aa381·R··R··S··L··A··K··P··A··F··P··G··G··M··G··I··L··P··P··V··Q· aa400
           cgaaggtcgttggcaaagcccgctttccccggcggcatgggcatcctgccgcctgtccaa


PAA14 aa401 G··L··P··L··A··G··I··C··W··A··A··R··G··S··*·    aa414
           ggccttccgcttgccggaatatgctgggcggcgcggggaagctag
```

Supplementary Figure 1. Frameshift alleles present in the international *P. aeruginosa* reference panel strains. **(A)** Frameshift present in the *exoY* allele of Mi162 (#30) and 5 additional strains (#13, #26, #29, #32, #42) after the codon for aa P242 (designated frameshift P242 in **Table 2**) resulting in a truncated protein of 248 aa. **(B)** Frameshift in the *exoY* allele of PA14 (#18) after the codon for aa F373 (designated frameshift F373 in **Table 2**) resulting in an extended protein of 414 aa.

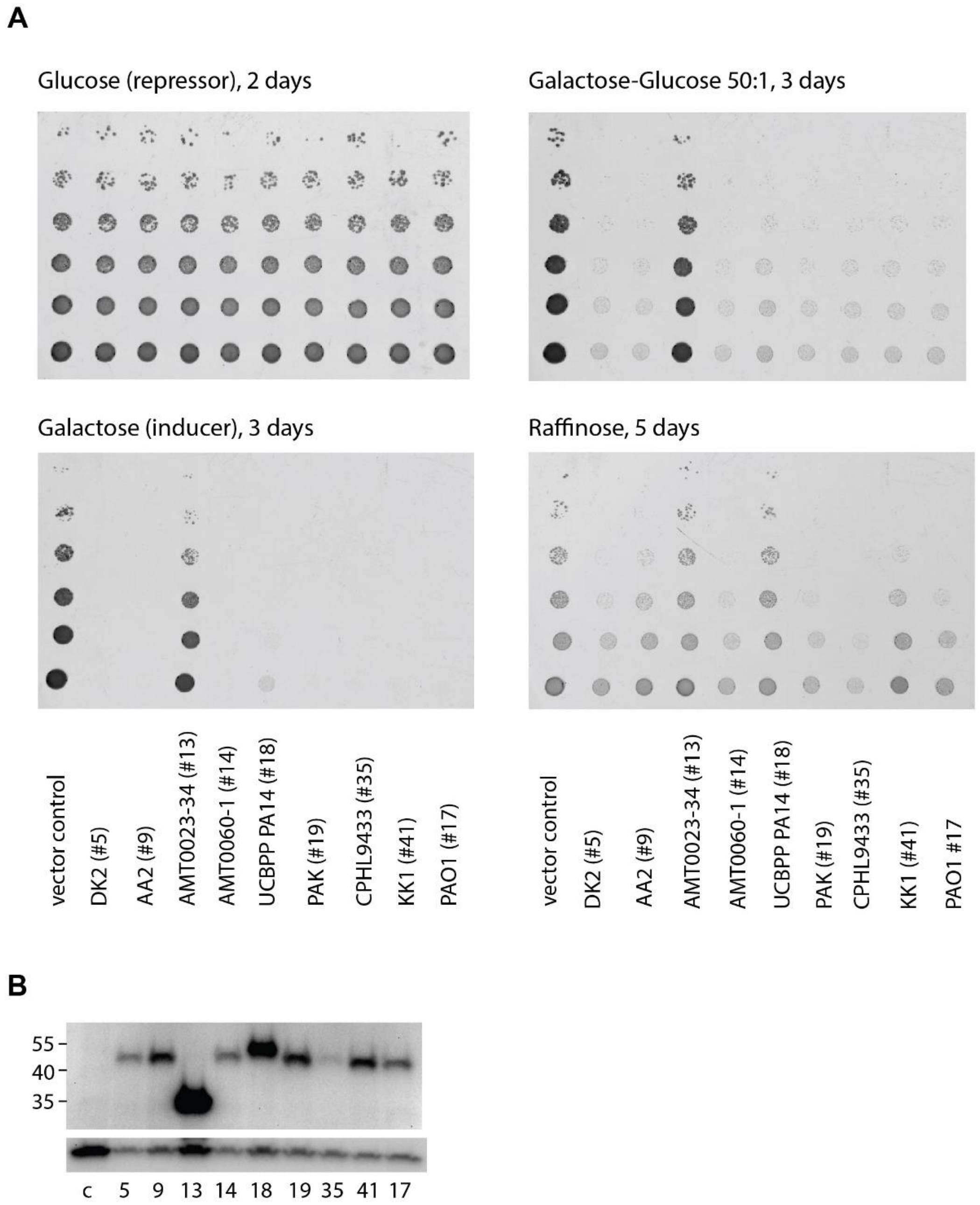

Supplementary Figure 2. Effect of SNPs present in *exoY* alleles of strains from the international *P. aeruginosa* reference panel on toxicity in *S. cerevisiae*. Alleles were expressed under the control of the GAL1 promoter in YEpGal555. **(A)** 5-fold serial dilutions of cell suspensions were prepared from overnight agar cultures by normalizing OD_{600} measurements, then spotted onto agar plates containing yeast nitrogen base without amino acids (Difco), supplemented with glucose (for repression of P_{Gal}), galactose (for high induction of P_{Gal}), a galactose-glucose mixture (for low levels of induction of P_{Gal}), or raffinose (for background induction levels of P_{Gal}) and incubated at 30 °C for 2-5 days as indicated. **(B)** Comparison of expression levels of ExoY by western blots with anti-myc (9B11, Abcam), upper panel and anti-RPS9 (loading control, a gift from Prof. S. Rospert, University of Freiburg), lower panel. c, vector control.

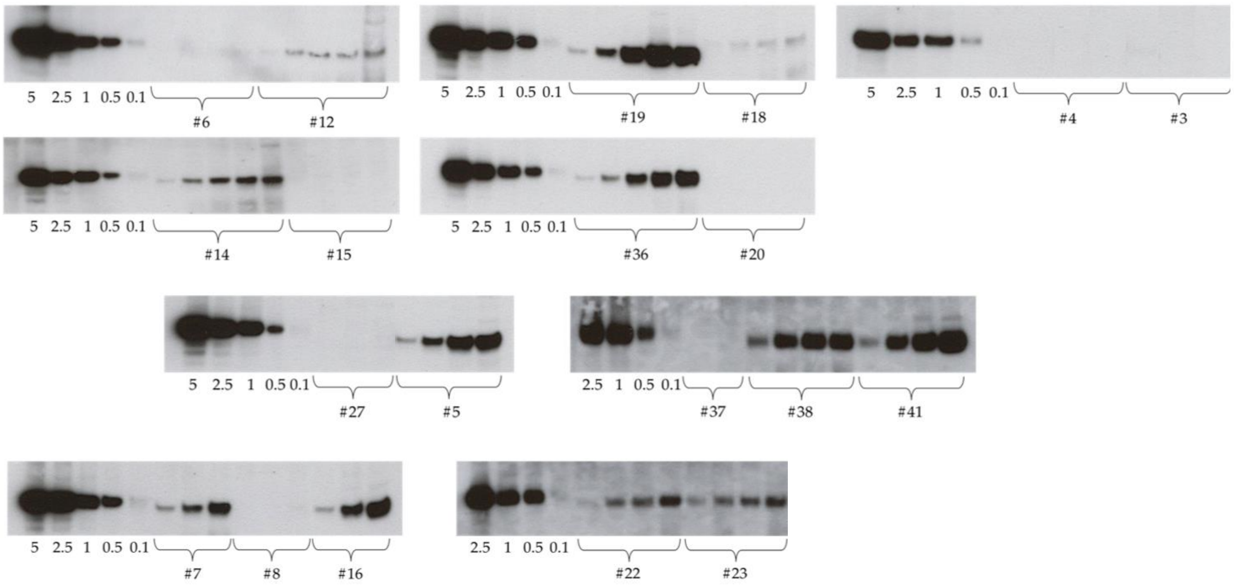

Supplementary Figure 3. Examples for western blots to detect ExoY in culture supernatants (11 μl). Recombinant ExoY was used as standard and loaded as indicated (between 0.1 and 5 ng). Primary rabbit polyclonal anti-ExoY antibodies against truncated ExoY (aa 26-223) at 1:2000 dilution and secondary ECL^{Tm} anti-rabbit-IgG horseradish peroxidase linked whole antibody from donkey (GE Healthcare) at 1:2500 dilution were used as detailed in Material and Methods.

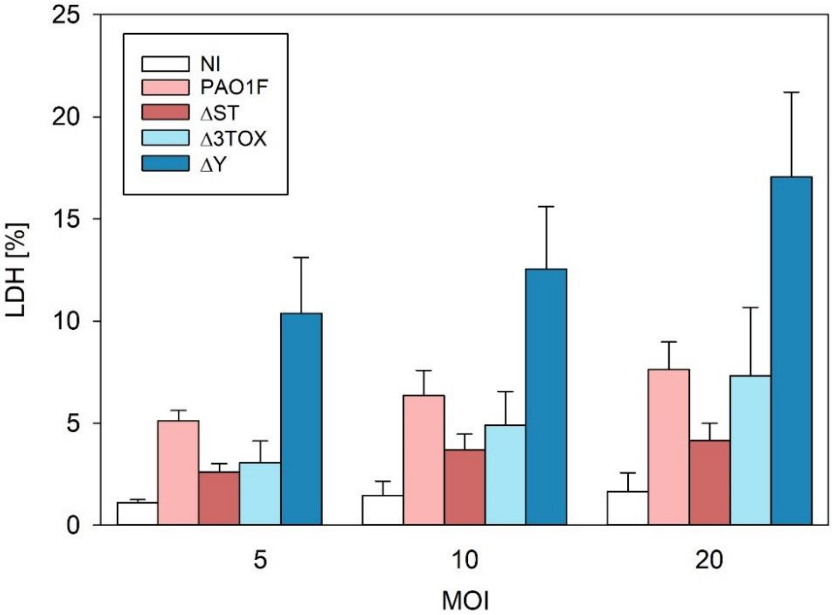

Supplementary Figure 4. LDH released in supernatants of NCI-H292 human lung bronchial epithelial cells following infection with *P. aeruginosa* for three hours at MOI 5, 10 and 20. The latter was chosen for further experiments to better detect the difference between cytotoxicity of the WT and the mutant strains. Cytotoxicity was assessed based on the amount of lactate dehydrogenase (LDH) released by epithelial cells into the culture supernatant measured as detailed in Materials and Methods (determined as % of total cell LDH). Mean values and standard deviation were calculated from three independent measurements taken using the CytoTox 96® Non-Radioactive Cytotoxicity Assay kit. The expression profiles of the T3SS effectors in the chosen set of strains were as follows: PAO1F, wild-type, *exoS*, *exoT* and *exoY* expression; Δ*ST*, only *exoY* expression; Δ*3TOX*, none of the T3SS effector genes expressed; Δ*Y*, *exoS* and *exoT* expression. NI, non-infected control.

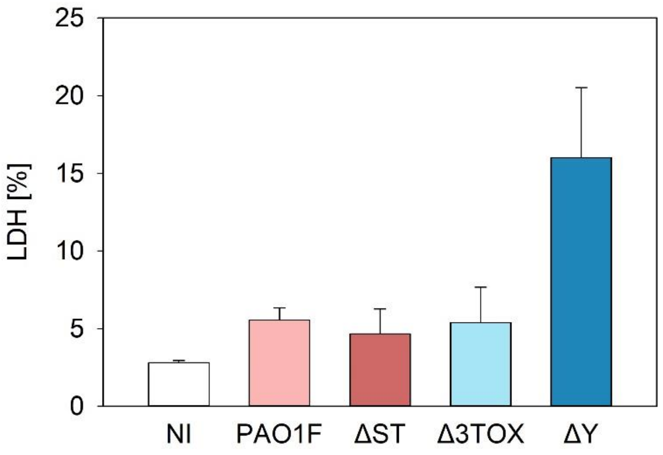

Supplementary Figure 5. Cytotoxicity of *P. aeruginosa* in A549 human alveolar epithelial cells increases upon *exoY* deletion that allows secretion of ExoS and ExoT as the only T3SS effector proteins. Cytotoxicity is assessed based on the amount of lactate dehydrogenase (LDH) released by epithelial cells into the culture supernatant measured as detailed in the Materials and Methods (determined as % of total cell LDH). Mean values and standard deviation were calculated from two independent measurements with 4 technical replicates each taken using the CytoTox 96® Non-Radioactive Cytotoxicity Assay kit. The expression profiles of the T3SS effectors in the chosen set of strains were as follows: PAO1F, wild-type, *exoS*, *exoT* and *exoY* expression; Δ*ST*, only *exoY* expression; Δ*3TOX*, none of the T3SS effector genes expressed; Δ*Y*, *exoS* and *exoT* expression. NI, non-infected control. One way ANOVA analysis with subsequent pairwise comparison (Turkey method) revealed significant differences ($P<0.001$) between all pairs that include ΔY and between PAO1F and NI ($P= 0.042$) (but not between other pairs).

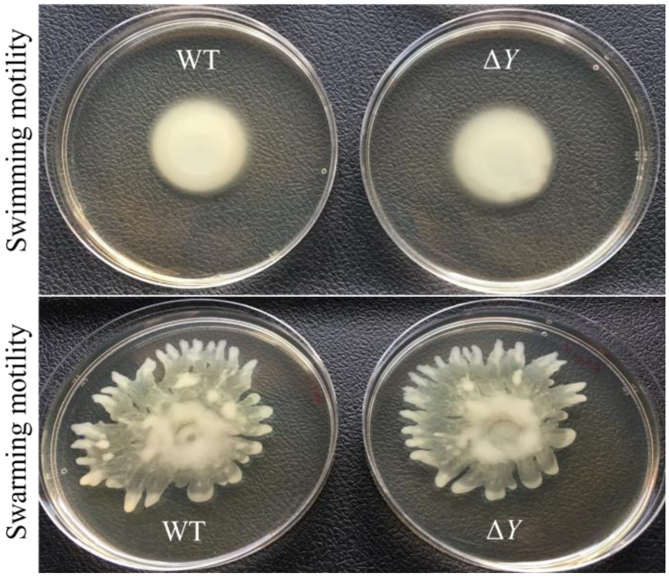

Supplementary Figure 6. Swimming and swarming motilities of the wild-type PAO1F strain and the *exoY* deletion mutant (Δ*Y*). Motility assays were performed as described in Materials and Methods, shortly, bacteria were inoculated onto 0.3% agar for swimming motility and 0.5% agar for swarming motility and plates were incubated at 37°C with aeration for 24 hours before taking photos. No difference was observed in these flagellum-driven motilities of the wild-type and the Δ*Y* strains. The experiment was performed 2 times and representative images are shown.
