# Supplementary material for: Prevalence of ExoY Activity in Pseudomonas aeruginosa Reference Panel Strains and Impact on Cytotoxicity in Epithelial Cells
Source: Front Microbiol. 2021 Oct 4;12:666097. doi: 10.3389/fmicb.2021.666097 (PMC8524455; doi:10.3389/fmicb.2021.666097)
Supplement: Supplementary file 2 [file Data_Sheet_2.pdf]

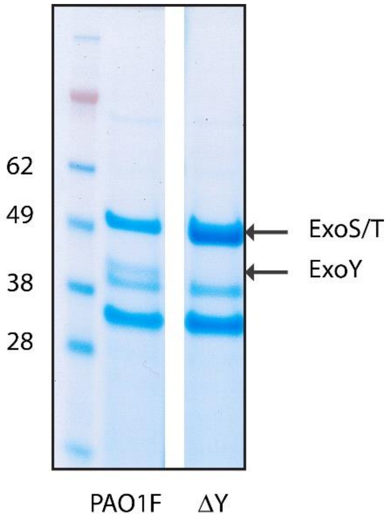

Supplementary Figure 7. Presence of ExoS, T and Y or Exo S and ExoT in bacterial culture supernatants of PAO1F and the DY mutant, respectively. Concentrated supernatants were analyzed by PAGE on 4-12 % NuPage gradient gels run in MES buffer and stained with Bio-Safe Coomassie G-250 (BIO - RAD). The identity of the proteins was determined by processing the bands cut from the gel by tryptic digests and analysis by tandem mass spectrometry (LC-MS/MS) revealed. A minimum of 30 % sequence coverage was achieved for protein identification.
